# A cost-effective and universal strategy for complete prokaryotic genome sequencing proposed by computer simulation

**DOI:** 10.1186/gb-2011-12-s1-p6

**Published:** 2011-09-19

**Authors:** Jingwei Jiang, Jun Li, Frederick C Leung

**Affiliations:** 1 School of Biological Sciences, Faculty of Science, The University of Hong Kong, China

## Background

Pyrosequencing techniques allow scientists to perform prokaryotic genome sequencing and achieve draft sequences within a few days. However, the sequencing results always turn out to contain several hundred contigs. A multiplex PCR procedure is then needed to fill all of the gaps and to link the contigs into one full-length genome sequence [1-10]. The full-length prokaryotic genome sequence is the gold standard for comparative prokaryotic genome analysis. This study assessed pyrosequencing strategies by using a simulation with 100 prokaryotic genomes.

## Results

Our simulation shows the following: first, a single-end 454 Jr Titanium run combined with a paired-end 454 Jr Titanium run may assemble about 90% of 100 genomes into <10 scaffolds and 95% of 100 genomes into <150 contigs; second, the average contig N50 size is more than 331 kb (Table 1); third, the average single base accuracy is >99.99% (Table 1); fourth, the average false gene duplication rate is <0.7% (Table 1); fifth, the average false gene loss rate is <0.4% (Table 1); sixth, the total size of long repeats (both repeat length >300 bp and >700 bp) is significantly correlated to the number of contigs (Table 4); and, seventh, increasing the read length of a pyrosequencing run could improve the assembly quality significantly (Table 1, 2, 3).

**Table 1:** Main average indices for different sequencing strategies for 100 genomes (400-bp read length)

ST	GCE (%)	SBE (%)	IDR (%)	FLT (%)	FDT (%)	CN	NB	SN
6xSE+10xPE	98.26971	0.004915	0.000364	0.310807	0.4678237	50.94	331136.7	3.64
10xSE+10xPE	98.30248	0.004265	0.000322	0.2626039	0.5629617	44.75	383793.6	3.51
15xSE+10xPE	98.32861	0.003293	0.000294	0.2518801	0.6041274	43.12	397060.7	3.48
20xSE+10xPE	98.35117	0.00227	0.000293	0.2307405	0.6301239	42.3	411169.2	3.66

ST: Sequencing Strategy; GCE: Genome Coverage Rage; SBE: Single Base Error Rate; IDR: Indel Error Rate; FLT: False Gene Duplication Rate; FDT: False Gene Loss Rate; CN: Contig Number; NB: Contig N50 Size (bp); SN: Scaffold Number.

**Table 2:** Main average indices for different sequencing strategies for 100 genomes (100-bp read length)

ST	GCE (%)	SBE (%)	IDR (%)	FLT (%)	FDT (%)	CN	NB	SN
6xSE+10xPE	98.06775	0.00498	0.000339	0.4892094	0.190552	72.11	209661.1	4
10xSE+10xPE	98.09051	0.003982	0.000324	0.4596817	0.180621	63.08	240424.9	3.8367
15xSE+10xPE	98.308065	0.004018	0.000322	0.4731213	0.1733068	61.77	241163.8	3.9184
20xSE+10xPE	98.10211	0.004231	0.000339	0.4754001	0.1754001	59.65	244658.8	3.7642

ST: Sequencing Strategy; GCE: Genome Coverage Rage; SBE: Single Base Error Rate; IDR: Indel Error Rate; FLT: False Gene Duplication Rate; FDT: False Gene Loss Rate; CN: Contig Number; NB: Contig N50 Size (bp); SN: Scaffold Number.

**Table 3:** Main average indices for different sequencing strategies for 100 genomes (200-bp read length)

ST	GCE (%)	SBE (%)	IDR (%)	FLT (%)	FDT (%)	CN	NB	SN
6xSE+10xPE	98.17144	0.003195	0.000334	0.4401864	0.2416131	61.15	253000.7	3.625
10xSE+10xPE	98.15661	0.004024	0.000317	0.4076573	0.2861061	54.33	290749.3	3.7188
15xSE+10xPE	98.16915	0.004743	0.000305	0.3916122	0.261398	53.47	301038.3	3.64
20xSE+10xPE	98.17177	0.004877	0.000309	0.409125	0.2509012	52.98	289864.6	3.6

**Table 4:** Linear regression results for 100 genomes, between the genome quality indicators and, for various read lengths, the number of repeats in the genome, the total repeat length of the genome and the percentage of the total repeat length of the genome

	Repeat length		Repeat length (>300)		Repeat length (>700)	
	
	R^2^	P-value	R^2^	P-value	R^2^	P-value
6XSE+10XPE, 400bp						
Number of Contigs	0.5657	2.2E-16	0.7842	2.2E-16	0.7948	2.2E-16
N50 size of Contigs	0.07932	0.00453	0.1107	0.00072	0.1114	0.0006918
Genome coverage	0.1298	0.0002314	0.2295	4.591E-07	0.2545	*.732E-08
SNP error rate	0.04819	0.0282	0.09175	0.002189	0.08484	0.003282
Indel error rate	0.002337	0.6329	0.04038	0.53	0.003728	0.5462
se gene duplication	0.2951	5.227E-09	0.2969	4.598E-09	0.2158	0.000001124
False gene loss rate	0.1978	0.00003553	0.338	2.264E-10	0.3408	1.827E-10
Number of Scaffolds	0.3363	2.565E-10	0.462	7.497E-15	0.4845	9.023E-16
10XSE+10XPE, 400bp						
Number of Contigs	0.4762	2E-15	0.6908	2.2E-16	0.7164	2.2E-16
N50 size of Contigs	0.05194	0.02258	0.09437	0.001878	0.09966	0.001377
Genome coverage	0.1185	0.0004542	0.2119	0.000001443	0.2358	0.000000305
SNP error rate	0.02702	0.1022	0.06257	0.01207	0.06363	0.01134
Inde1 error rate	0.0006153	0.8065	0.001432	0.7085	0.001119	0.7411
se gene duplication	0.3133	1.414E-09	0.324	6.457E-10	0.2426	1.936E-07
False gene loss rate	0.1232	0.0003429	0.2021	0.000002708	0.1943	0.000004425
Number of Scaffolds	0.2813	1.384E-08	0.4074	9.141E-13	0.4424	4.417E-14
15XSE+10XPE, 400bp						
Number of Contigs	0.453	1.709E-14	0.6676	2.2E-16	0.7008	2.2E-16
N50 size of Contigs	0.01038	0.3131	0.07265	0.006691	0.07771	0.004978
Genome coverage	0.1149	0.0005616	0.02068	0.00002001	0.2323	3.837E-07
SNP error rate	0.0001226	0.913	0.0004724	0.83	0.0002939	0.8656
Inde1 error rate	0.0001226	0.913	0.0004724	0.83	0.0002939	0.8656
se gene duplication	0.3217	7.638E-1C	0.3318	3.595E-10	0.2468	1.465E-07
False gene loss rate	0.1541	0.00005366	0.2604	5.834E-08	0.2642	4.519E-08
Number of Scaffolds	0.4023	1.399E-12	0.5996	2.2E-16	0.5878	2.2E-16
6XSE+10XPE, 400bp						
Number of Contigs	0.448	2.696E-14	0.6554	2.2E-16	0.6869	2.2E-16
N50 size of Contigs	0.05142	0.02328	0.09641	0.001666	0.1006	0.001301
Genome coverage	0.1152	0.000551	0.2076	0.000019	0.2338	3.467E-07
SNP error rate	0.2124	0.000001398	0.3199	8.7E-10	0.3315	3.678E-10
Inde1 error rate	0.00001646	0.968	0.00016	0.9006	0.00006389	0.937
se gene duplication	0.3492	9.627E-11	0.3761	1.182E-11	0.2922	6.453E-09
False gene loss rate	0.1163	0.000515	0.2011	0.000002892	0.1938	0.000004569
Number of Scaffolds	0.3125	1.495E-09	0.458	1.09E-14	0.4898	5.431E-16

## Conclusions

A single-end 454 Jr run combined with a paired-end 454 Jr run is a good strategy for prokaryotic genome sequencing. This strategy provides a solution to producing a high-quality draft genome sequence of almost any prokaryotic organism, selected at random, within days. It could be the first step to achieving the full-length genome sequence. It also makes the subsequent multiplex PCR procedure (for gap filling) much easier, aided by the knowledge of the orders/orientations of most of the contigs. As a result, large-scale full-length prokaryotic genome-sequencing projects could be finished within weeks.
